# Standing on the Shoulders of the Giants: Dr. Alain Fischer

**DOI:** 10.46989/001c.87882

**Published:** 2023-09-16

**Authors:** Mohamad Mohty, Alain Fischer

**Affiliations:** 1 Sorbonne University, AP-HP, INSERM UMRs938, Paris, France; 2 Service d’Hématologie Clinique et de Thérapie Cellulaire, Hôpital Saint Antoine, AP-HP, Paris, France; 3 Pediatric Hematology-Immunology and Rheumatology Department, Hôpital Necker-Enfants Malades, AP-HP Centre Université de Paris, Paris, France; 4 Institut Imagine, INSERM UMR 1163, Paris, France; Collège de France, Paris, France.

In a letter to Robert Hooke in 1675, Sir Isaac Newton made his most famous statement: “if I have seen further, it is by standing on the shoulders of giants.” This initiative of the International Academy for Clinical Hematology (IACH) aims to celebrate the achievements of leading experts and investigators whose work and research have helped to significantly advance the field of clinical hematology and establish the milestones and foundations of modern clinical hematology. This report represents a transcript of the interview given by Dr Alain Fischer (AF) (**Figure 1**) on the 4th of May 2023, who responded to a series of questions asked by Dr Mohamad Mohty (MM).

**Figure 1.**
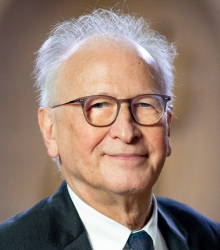
Dr. Alain Fischer

MM: Our honoree today is Professor Alain Fischer, who has been a pioneer in the field of genetic disorders bringing revolutionary curative gene therapies for orphan and ultra-rare diseases in children and young adults. Thank you for joining this series, and we are very privileged and honored to have you with us. Can you tell us something about your early childhood and early educational experience that might have played a role in your future career and selection of the scientific and medical field?

AF: Good evening from the Imagine Institute in Paris. I am honored and feel a little embarrassed by the term ‘giant,’ but anyway, I am pleased to discuss what I have tried to do during my career with you. As a child, I don’t think there is anything special to say. When I was at primary school, I was pretty serious, but then at senior school as a teenager, like many boys, I didn’t work that much and was not interested in schooling. I became better when I started my medical studies. Why did I start in medicine? I’m not sure that I know, I have no family background in medicine, my children are doing completely different things. My father was living in Hungary during the period following the First World War. He was Jewish, and at that time, Jews were being persecuted he had the idea of doing medicine, but that was not possible due to the numerous clauses established against the Jews of Hungary so perhaps this was part of what determined me to go into medicine.

MM: Was there a particular teacher in High School or later at university that you thought of as exemplary and wanted to follow?

AF: Well, yes, but that came later, and there are a few people who I should mention. The first one was not very well known in hematology as he was a pediatrician called Pierre Royer at the Necker-Enfants Malades hospital where I am working. He was absolutely instrumental in the development of research in pediatrics. I had him as a teacher in the Faculty, and I was a resident in his department when I trained as a pediatrician. He was extremely smart, and his vision was that medicine had to be associated with research in a way that we had to do the best we could to advance medicine I was very impressed by his way of thinking and broad-mindedness. He not only looked at what we know about medicine but asked key questions about what we don’t know and how we can approach what we do not know. Another one was Maxime Seligmann, an immunologist at the Saint-Louis Hospital in Paris; again, I was a resident in his department. This man was a pioneer in clinical immunology; for instance, he did fantastic things in determining the presence of antinuclear antibodies in lupus. Again, I was very impressed by the way he was approaching medicine, not only concerning patient care but in the way he used his scientific mind in asking the questions. There are three other people I would like to mention, Claude Griscelli my mentor whom I succeeded in pediatric immunology/hematology at Necker-Enfants Malades. He created, in France, the field of the study of genetic disorders of the immune system in which I strived afterward; he was really the one who started the field.

Later on, I did a post-doc in the UK at University College London (UCL), and two people had a major impact on my research: Marc Feldmann, very well known for his work on anti-TNF treatment of inflammatory and autoimmune diseases, and Peter Beverley, another very smart immunologist and so I learned basic immunology with them and I consider myself more of an immunologist than a hematologist although I am on the frontier between them. Those two people taught me how to think and work in immunology.

MM: So, you trained as a pediatrician and so my question is, why pediatrics? Within pediatrics, you switched progressively to this topic of immune deficiencies, so what attracted you to move into such a narrow and orphan field?

AF: I know the answer to this because during my medical studies, actually by chance, I went for a short period to the unit that Claude Griscelli was developing. This was the beginning of the study of such diseases, in France, following Bob Good in the United States (US) and a few others. I was amazed by the interest in this field and in the way he was taking care of these very sick patients who were children of course and were protected not only physically but also psychologically. I thought then that this was exactly what I wanted to do. So, if I wanted to work in that area, I needed to train in pediatrics first, which explains my pathway to this narrow but exciting field.

MM: Absolutely, I mentioned narrow but without any negative connotation! You were appointed Full Professor at age 39, which is quite unusual, as a Professor in Pediatric Immunology here in Paris. What was the status of the research on genetic disorders during this period, and how did you decide that you needed to move things forward because I believe that things were stagnating.

AF: Actually, I came at the right time, I was very lucky, and I was not the only one. Many people from the same generation interested in genetics came at a time when the technology had been developed to study the genome. I remember very well during my post-doc, as I mentioned previously, with my friend Peter Beverley, a talk by a British-Australian geneticist on the very first efforts in trying to map genes, in the early 80s. It came to my mind that, wow, that’s potentially very exciting and we may approach these genetic diseases that were described phenotypically, sometimes quite precisely, not always but some were very precise descriptions that started in the 50s, 60s, 70s and there were more and more phenotypic descriptions with the immunological tests that became available but of course we lacked the cause because we did not know the genes. The very first gene that was found in 1984 to be associated with primary immunodeficiency was the gene for adenosine deaminase, one form of severe combined immunodeficiency (SCID). At the same time, one of the genes for chronic granulomatous disease was deciphered in the US, so that was the very beginning. Obviously, there was an opening field and many people jumped into that field in different areas of genetics. I arrived at the right time with my colleagues at Necker-Enfants Malades hospital to study the genetic basis of many of these primary immunodeficiencies.

MM: Can I ask you to go into more detail about the different interactions you had with these colleagues you mentioned, who were really pioneers, and when was the turning point that made you want to apply this to the first gene therapy? In 2023, discussing gene therapy is quite common, but 25 years ago, I don’t think there were too many believers in it.

AF: No, of course not, but that was a natural consequence of the studies we had performed in the 80s and 90s in trying to decipher the genetic causes of several of these immunodeficiencies. One of them that we were working on with Geneviève de Saint-Basile, a smart scientist, still active here, was to look at the cause of a disease named X-linked SCID disease, the SCID diseases are those where the T lymphocyte is absent, leading to early death in the absence of allogeneic transplantation. This latter treatment had been available since 1968, but it was not always successful. So, we mapped the locus on the X chromosome and another group were actually first to clone the gene but that’s OK. We were still interested in studying that particular disease mechanism and why there was an absence of T lymphocytes and NK (natural killer) lymphocytes in such patients and Geneviève went to study a special unique case, which was the key point that led us to gene therapy. This was a boy from Germany referred to us because his doctors could not understand that even though he had the mutation that should have led to a SCID, didn’t have an SCID. Geneviève showed that the boy’s blood had T lymphocytes that reverted from the mutation, by chance, and led to a significant proliferation of T lymphocytes and the child didn’t die and had a much better outcome with only a mild infection. What happened in this child we can call natural gene therapy and if it works in a single event, a single cell being corrected by chance leading to the development and proliferation of many T-cell precursors and taking into account the very long lifetime of these cells, maybe we could engineer to do slightly better than correcting one cell, and even with the technology available at that time, there was a chance of a cure. This was how we decided to embark on a gene therapy program at a time when there had been many attempts but no success. The technology was improving and several groups all over the world were contributing with retroviral vectors, and methodology to increase the transduction rate of such cells. All of this knowledge was collected from these groups and a program of gene therapy for X-linked SCID began, around 1995-96. This was based on the study of these revertants.

MM: But then after the first successful attempts you monitored some of these patients and I remember the story of the acute leukemia which I believe was a big shock to some people and put a full stop on this field, but you were a little stubborn and persisted to understand how this happened and how it could be corrected.

AF: Yes, we restarted the X-SCID clinical trials we had performed with Marina Cavazzana and Salima Hacein-Bey-Abina, they were initiated in the first trimester of 1999. By the way, I remember the first patient we injected into the central line, we were very apprehensive about what would happen, that was in March of that year; today he is 25 years old and is doing well. He has T-cells and is living normally. Three years later came the first case of leukemia and then a few more which was a shock both for us and the families. Unfortunately, the first patient who developed leukemia died but the others were rescued quite easily by treatment, even corticosteroids were effective in getting rid of the blasts and they had chemotherapy of course. Interestingly, following the chemotherapy, these patients still had transduced T lymphocytes and still have today, 20 years later and are doing well.

There were two possibilities after these leukemias occurred in these trials, either to stop the program or modify it. Given the efficacy and recovery of T-cell function in these patients we decided to build on the positive aspects and try and fix the negative aspects. In collaboration with a number of people in Germany, the UK, and US, we embarked on trying to understand what happened, which we did in a few years. Something that was not known at the time of the first clinical trial because we did not have the sequence of the human genome which came two years later, we realised that the vector was integrating within genes and not randomly as was thought, and within active genes among hematopoietic progenitor cells there are a few oncogenes, notably one named LMO2 which is very well known by specialists in T-cell leukemia. We realised that in some of these patients, such an integration led to trans-activation of the oncogene. The solution came after discussion with a number of people and instead of using what are now called the ‘first generation’ of vectors where we were using the long terminal repeats (LTR]), extremities of the retrovirus, that drive the expression of the gene because there is a strong enhancer in the LTR. We thought we should get rid of this enhancer to avoid trans-activation and instead, use a promoter with a much lower enhancer activity. So, this was done, with collaboration, notably with Adrian Thrasher at UCL. It took time but a new clinical trial began in 2009 with this so called ‘second generation’ vector. This worked in terms of efficiency and without leukemia.

MM: Fascinating! You are definitely the father of gene therapy.

AF: There are many fathers ….. and mothers!

MM: OK, so let’s say you are a unique father, or even grandfather, although you are still very young. You mentioned the names of some of your collaborators and so now I’d like to ask a broader question about all of these physicians, doctors, scientists that you have trained with or that trained with you. Did you see yourself playing a role in influencing their careers and pushing them in the direction of this specific field?

AF: I hope so. An important part of the career of a university professor is to teach younger people and help them to develop their own projects, whether in gene therapy or other fields, I don’t care, provided that they do it very well. I’m quite proud to see a few of them today doing a fantastic job. For instance, in Canada, two people, Nada Jabado who has become a world specialist in pediatric oncology, so not doing gene therapy but doing extremely well in understanding the complex genetic mechanism of neural tumours. Also, Elie Haddad in Canada and James di Santo in Paris and Jean Laurent Casanova in New York. I am proud that some people who worked with me have done very well in their own careers, totally independently of me. Perhaps this is what I am most proud of.

MM: Well, you can be proud because actually you have trained dozens of colleagues in this field and you were the initiator of the Inherited Disorders and Inborn Errors Working Party of the European Society of Blood and Marrow Transplantation (EBMT). A few minutes ago, you mentioned your first patient, with whom you are still in contact and so you are aware that this patient is alive and well 25 years later. I would love to hear about your relationship with your patients and their families and how this has influenced the research questions that you have chosen to ask in your career.

AF: One of the pleasant aspects of my career was to maintain strong relations with patients. There is another SCID patient who received an allogeneic bone marrow transplant when I was a resident in 1977. I am still in contact with this guy who is now 46 years old. It is fantastic to maintain relationships with these patients. I have ceased clinical activity except for those patients who had gene therapy, I’m still seeing them once or twice a year. This is important and fantastic to see how their lives evolve. They ask many questions which may drive some of our research and in fact one part of the research that I am continuing with people here like Bénédicte Neven, is to understand what is going on in the immune system of someone who received a transplant 40 or 50 years ago, or gene therapy 20 or 25 years ago. This is something we continue to do which is based on these patients. In fact, this morning I called one and said we had a new program, a clinical trial, and it would be nice if they could have a skin biopsy! We have quite a special relationship and it is exciting both in terms of human interaction and scientifically. I know these guys pretty well except for one who is living in Alaska!

MM: Well, we can go together if you wish. I can arrange an outpatients’ clinic there.

AF: Most of them I know well and the grandmother of one patient knows that I like good food and her grandson brings me some homemade confiture.

MM: Very nice, this is fantastic. On a personal level, if you’ll allow me, can you talk about your family and their contribution and how they view your career and dedication to your patients.

AF: My wife is a hematologist in the field of thrombosis. She has retired now but was the head of the hematology department at the Hôpital Européen Georges Pompidou, a new hospital in Paris. So, she had her own career despite the fact that we had two boys, one of whom is doing research in mathematics and the other is shooting documentaries, so both far away from medicine.

MM: We can take this one with us to Alaska because the landscape is beautiful.

AF: Well, he is more interested in people than landscapes, people who are suffering mentally or socially. I am very glad that despite the fact that I had to work a lot and so was not present in the family very much, my wife was a good manager and had a good career. Both my wife’s parents and mine were also living in Paris so we were lucky as they looked after the children a lot and the children liked going to their grandparents. This enabled both of us to carry on with our careers. I have three granddaughters and maybe they will do something to do with science, but I don’t know, they are too young yet.

MM: We will follow them carefully. Another question. If you look back over the last 30 years or so, is there anything that you would have preferred to have done differently. Do you have any regrets or are you happy with the way things have gone?

AF: I don’t have so many regrets, as I’ve said before, I came at a good time and was very lucky. You mention the Imagine Institute, which is a relatively new research institute, opened in 2014, where we have great facilities to carry out research on genetic diseases including gene therapy. Maybe one regret is that it would have been better if it had opened 10 years earlier, but I can’t complain as I’ve had a wonderful working environment compared with many of the institutions of colleagues in France, I have been privileged.

MM: I have one last question. It looks like gene therapy is the future whether in hematology, malignant or non-malignant disorders. We are manipulating the genes of almost everything these days. What are your hopes for this field and what advice have you for the younger generation of medical student or scientist embarking in this field?

AF: I would say that gene therapy is fine and there are new developments in genome editing for instance, with great promise technologically speaking, with many more applications but don’t forget that gene therapy is only one aspect of future medicine. Of course, I am not saying that gene therapy will not provide more cures of genetic and non-genetic diseases but there are other strategies and if you can do something else that is smart or smarter, then do it and don’t restrict your mind. Let’s go where the science is. To be successful you have to understand the basics of the disease, its pathophysiology, this is the first step. If you try to do gene therapy without this understanding, it’s very unlikely that you will succeed. This is obvious but maybe it should be reiterated from time to time. It is the first step on which you can build therapy and then you can decide if gene therapy is the best approach or should you try something else, depending on the cases.

MM: You are definitely advocating the physician scientist.

AF: Yes of course. It is more complicated for young people today than it was for my generation because science *per se* has progressed and there are more things to understand and learn. The same is true for medicine but I believe we need as many physician scientists as possible in our university hospitals to be able to advance.

MM: Well, we’ve reached the end of this interview, would like to add anything further?

AF: Maybe to say that young people should of course respect their teachers and listen to them, but they should also criticise them gently and ask questions because it is only by asking questions that you will find your own way. Don’t just listen to old guys like me without questioning them.

MM: On behalf of dozens of patients and their families, not only in France but across the globe, I would like to convey our gratitude and admiration for the giants and the old guys like yourself, Professor Fischer, it has been a true pleasure to have this interview with you today and I hope to catch up with you at some point. I hope that everybody has enjoyed this broadcast.

AF: Thank you very much.

## ETHICAL APPROVAL

Not applicable.

## CONSENT TO PARTICIPATE/INFORMED CONSENT

Not applicable.

## CONSENT FOR PUBLICATION

Not applicable.

## COMPETING INTERESTS/CONFLICT OF INTEREST

Not applicable.

## AUTHORS’ CONTRIBUTION – CREDIT TAXONOMY

Conceptualization: Mohamad Mohty and Alain Fischer (Equal). Writing – original draft: Mohamad Mohty (Lead). Writing – review & editing: Mohamad Mohty and Alain Fischer (Equal).

